# Association of Open vs Robot-Assisted Radical Cystectomy With Mortality and Perioperative Outcomes Among Patients With Bladder Cancer in Sweden

**DOI:** 10.1001/jamanetworkopen.2022.8959

**Published:** 2022-04-28

**Authors:** Ashkan Mortezavi, Alessio Crippa, Maria Ioanna Kotopouli, Olof Akre, Peter Wiklund, Abolfazl Hosseini

**Affiliations:** 1 Department of Urology, University Hospital Basel, Basel, Switzerland; 2 Department of Medical Epidemiology and Biostatistics, Karolinska Institutet, Stockholm, Sweden; 3 Department of Molecular Medicine and Surgery, Section of Urology, Karolinska Institutet, Stockholm, Sweden; 4 Division of Biostatistics, Institute of Environmental Medicine, Karolinska Institute, Solna, Sweden; 5 Department of Pelvic Surgery, Department of Pelvic Cancer, Karolinska University Hospital, Stockholm, Sweden; 6 Department of Urology, Icahn School of Medicine at Mount Sinai, New York, New York

## Abstract

**Importance:**

Mortality rates resulting from bladder cancer have remained unchanged for more than 30 years. The surgical community has put hope in robot-assisted radical cystectomy (RARC) with intracorporeal urinary diversion (ICUD) in an effort to improve surgical outcomes and bladder cancer survival without strong supporting evidence.

**Objective:**

To evaluate perioperative, safety, and survival outcome differences between RARC with ICUD and open radical cystectomy (ORC).

**Design, Setting, and Participants:**

This nationwide population-based cohort study used data from the Swedish National Register of Urinary Bladder Cancer and population-based Cause of Death Register, which includes clinical information on tumor characteristics, treatment, and survival and covers approximately 97% of patients with urinary bladder cancer in Sweden. All patients who underwent radical cystectomy for bladder cancer in any hospital between January 2011 and December 2018 were included. Follow-up data were collected until December 2019. Data analysis was conducted from June to December 2020.

**Exposures:**

RARC or ORC.

**Main Outcomes and Measures:**

The main outcomes were all-cause and cancer-specific mortality between RARC and ORC, compared using propensity score matching. Secondary outcomes were differences in perioperative outcomes after the different surgical approaches.

**Results:**

Throughout the observation period, 889 patients underwent RARC and 2280 patients underwent ORC at 24 Swedish hospitals. The median (IQR) age was 71 (66-76) years and 2386 patients (75.3%) were men. After a median (IQR) follow-up of 47 (28-71) months, the 5-year cancer-specific mortality rates were 30.2% (variance, 1.59) for ORC and 27.6% (variance, 3.12) for RARC, and the overall survival rates were 57.7% (variance, 2.46) for ORC and 61.4% (variance, 5.11) for RARC. In the propensity score–matched analysis, RARC was associated with a lower all-cause mortality (hazard ratio, 0.71; 95% CI, 0.56-0.89; *P* = .004). Compared with ORC, RARC was associated with a lower estimated blood loss (median [IQR] 150 [100-300] mL vs 700 [400-1300] mL; *P* < .001), intraoperative transfusion rate (odds ratio [OR], 0.05; 95% CI, 0.03-0.08; *P* < .001), and shorter length of stay (median [IQR], 9 [6-13] days vs 13 [10-17] days; *P* < .001), and with a higher lymph node yield (median [IQR], 20 [15-27] lymph nodes vs 14 [8-24] lymph nodes; *P* < .001) and 90-day rehospitalization rate (OR, 1.28; 95% CI, 1.02-1.60; *P* = .03). The RARC group, compared with the ORC group had lower risk of Clavien-Dindo grade III or higher complications (OR, 0.62; 95% CI, 0.43-0.87; *P* = .009).

**Conclusions and Relevance:**

These findings suggest that compared with ORC, RARC with ICUD was associated with a lower overall mortality rate, fewer high-grade complications, and more favorable perioperative outcomes.

## Key Points

**Question:**

What are the perioperative, safety, and survival outcomes of robot-assisted laparoscopic radical cystectomy (RARC) with intracorporeal urinary diversion compared with open radical cystectomy (ORC)?

**Findings:**

This cohort study included 3169 patients undergoing cystectomy for bladder cancer between 2011 and 2018 in Sweden. After propensity score matching, patients who underwent RARC had lower overall mortality rate, fewer high-grade complications, and more favorable perioperative outcomes compared with those who underwent ORC.

**Meaning:**

These findings suggest that in spite of the technically complex nature of the fully intracorporeal approach, nationwide implementation is feasible, with beneficial outcomes for patients with bladder cancer.

## Teaser

This cohort study compares perioperative, safety, and survival outcomes between robot-assisted radical cystectomy and open radical cystectomy among patients with bladder cancer in Sweden.

## Introduction

Radical cystectomy with pelvic lymph node dissection (PLND) is considered the criterion standard treatment for muscle invasive bladder cancer and selected patients with high-risk nonmuscle invasive bladder cancer^1^ and is associated with high rates of perioperative morbidity and mortality.^2^ Minimally invasive strategies have gained popularity because of their potential to reduce surgical morbidity and shorten hospital length of stay (LOS). Since its introduction in 2003, robot-assisted laparoscopic radical cystectomy (RARC) has been gradually adopted as a surgical option with the goal to improve perioperative outcome and survival.^3^

Available studies investigating the perioperative safety and oncological efficacy of RARC compared with open radical cystectomy (ORC) are primarily small trials at academic institutions with short follow-up time.^4^ Additionally, the diversion technique has been almost exclusively limited to an extracorporeal hybrid approach (ECUD), leaving the question concerning the risks and benefits of a fully minimal-invasive intracorporeal technique unanswered.

Sweden has been an early adopter of RARC with intracorporeal urinary diversion (ICUD), with introduction of the technique in routine clinical practice in 2004.^5^ The procedure was implemented at multiple national sites in the subsequent years providing an opportunity to perform a broad-based analysis of its long-term efficacy. In this study, we used nationwide population-based data to compare perioperative and oncological outcomes after RARC vs ORC in Sweden over a period of 8 years.

## Methods

This cohort study was approved by the Stockholm research ethics board. Because this study used registry-based data, informed consent was not required, per Swedish regulations.^6^ This study is reported following the Strengthening the Reporting of Observational Studies in Epidemiology (STROBE) reporting guideline.

### Data Source

We retrieved data from the Swedish National Register of Urinary Bladder Cancer (SNRUBC), which includes clinical information on tumor characteristics and treatment and covers approximately 97% of patients with urinary bladder cancer in Sweden.^7,8^ In the database, cause and date of death were retrieved from the population-based Cause of Death Register using the national registration number assigned to all Swedish residents.^9^

### Patient Population and Covariates

We included all patients who underwent RC for bladder cancer in any hospital between January 2011 and December 2018 using *International Statistical Classification of Diseases and Related Health Problems, Tenth Revision* (*ICD-10*) code C67.9. Patients with metastatic disease before surgery were excluded. Patient covariates included age, sex, body mass index (BMI; calculated as weight in kilograms divided by height in meters squared), American Society of Anesthesiologists (ASA) score, status of prior abdominal or pelvic surgery or radiotherapy, clinical T- and N-stage, and receipt of neoadjuvant chemotherapy (NAC). Hospital volume was calculated as the mean annual caseload of RCs at a given institution during the study period. All procedures were either classified as ORC or RARC.

### Outcomes

Our primary aim was to assess all-cause and cancer-specific mortality after RARC and ORC. Secondarily, we aimed to study differences in perioperative parameters, postoperative complications, and length of stay after the different surgical approaches. Intraoperative parameters included the performance of PLND, performance of urethrectomy, diversion type, estimated blood loss (EBL), intraoperative transfusion rate, and operation time. Diversion type was classified as ileum conduit, orthotopic bladder substitution (OBS), continent cutaneous urinary diversion, and others. RARC procedures converted to open surgery remained in the RARC cohort. Postoperative data included 90-day complications by type categorized using the modified Clavien-Dindo (CD) classification,^10^ reoperation rate, LOS, 90-day rehospitalization rate, pathological T-stage, number of removed lymph nodes (LNs), and nodal status. Death from bladder cancer was defined as *ICD-10* code C67.8, C67.9, C68.9, or D41.4 as underlying death cause. Starting date of the study was the date of RC and last date of the study was date of death, emigration, or December 31, 2019, whichever happened first.

### Statistical Analysis

First, we compared baseline characteristics of patients undergoing ORC vs RARC. Differences between the groups were statistically assessed using the Pearson χ^2^ for categorial variables and Mann-Whitney *U* test for continuous variables.

Second, to account for possible selection bias, observed differences in baseline characteristics between patients who received ORC vs RARC were addressed using propensity score (PS) matching. Specifically, a logistic regression PS model for the type of the surgical approach (ORC vs RARC) was created, and the following variables were included in the model: age, BMI, ASA score, prior surgery or radiotherapy, clinical T- and N-stage, NAC, annual hospital volume, and pathological T-stage. Fixed ratio (1:2) nearest-neighbor matching was performed with a 0.2-width caliper of the SD of the logit of the PS.^11^

Third, descriptive statistics were used to summarize data for the PS-matched groups and balance between covariates was assessed by using the standardized mean difference (SMD). Primary and secondary outcomes were compared in the PS-matched cohort. Results are presented as median (IQR) or frequency (percentage). Categorical end points were assessed using logistic regression accounting for clustering in a fixed effects model. Cumulative incidence functions for cancer-specific and other-cause mortality were estimated nonparametrically with the Aalen-Johansen estimator and compared between ORC and RARC using the Gray test.^12^ Overall survival (OS) curves were derived as 1 – all-cause mortality and compared between surgical groups with the log-rank test. We compared cancer-specific mortality, other-cause mortality, and all-cause mortality in the RARC vs the ORC groups by using Cox regression models using robust variance estimation to take into account the correlation of matched individuals. We stratified the estimated Cox regression models by hospital, allowing separate baseline hazard functions for each hospital to further capture between-hospital variability. We evaluated the robustness of study results to possible unmeasured confounders by computing the *E*-values.^13^ We further performed 2 different sensitivity analyses: in the first analysis we included year of surgery as matching variable for PS matching. For the second analysis, we excluded all low-volume hospitals (<25 RCs per year) before PS matching to further address the potential confounding bias of surgical experience. For the subgroup analysis of the population not receiving NAC, these patients were excluded from the PS-matched cohort.

Statistical analyses were performed by using SPSS version 26.0 (SAS Institute) and R version 4.1.2 (R Project for Statistical Computing) statistical software. All tests were 2-sided, and *P* < .05 was considered statistically significant. Data analysis was conducted from June to December 2020.

## Results

### Baseline Characteristics and PS Matching

Between January 2011 and January 2019, RC for bladder cancer in nonmetastatic disease was performed in 3169 patients at 24 different hospitals in Sweden (RARC at 8 centers, including 52 patients [5.8%] with ECUD and 837 patients [94.2%] with ICUD). The median (IQR) age was 71 (66-76) years, and 2386 (75.3%) were men. The studied population had a median (IQR) BMI of 26 (23-28). A total of 1760 patients (55.5%) were classified as ASA II, and 967 patients (30.5%) were classified as ASA III. Nearly 1 in 5 patients (586 patients [18.5%]) had prior abdominal surgery or radiation to the pelvis. Baseline oncological parameters showed muscle invasive disease (≥T2) in 2294 patients (72.4%), and 1048 patients (33.1%) received NAC. Baseline characteristics of the entire cohort stratified for 2280 patients who underwent ORC (71.9%) and 889 patients who underwent RARC (28.1%) are displayed in Table 1. Survival curves and univariate Cox regression analysis are presented in eFigure 1 and eTable 1 in the Supplement. The PS-matched cohort included 2428 patients (1554 patients [64.0%] in the ORC group and 874 patients [36.0%] in the RARC group). Following matching, all covariates were comparable between the ORC and RARC group (absolute SMD < 0.1) (Table 1). The PS distributions for the groups both before and after matching confirming the adequacy of the model are shown in eFigure 2 in the Supplement.

**Table 1.** Baseline Patient Characteristics Overall and After Propensity Score Matching

Characteristic	Before propensity matching				After propensity matching			
	No. (%)		SMD	*P* value	No. (%)		SMD	*P* value
	ORC (n = 2280)	RARC (n = 889)			ORC (n = 1554)	RARC (n = 874)		
Sex								
Men	1713 (75.1)	673 (75.4)	−0.01	.74	1160 (74.6)	660 (75.5)	−0.02	.67
Women	567 (24.9)	216 (24.3)			394 (25.4)	214 (24.5)		
Age, median (IQR), y	71 (66 to 76)	71 (65 to 76)	−0.07	.12	71 (65 to 76)	71 (65 to 76)	0.003	.80
BMI								
Median (IQR)	26 (23 to 28)	26 (23 to 28)	0.21	.37	26 (23 to 28)	26 (23 to 28)	−0.03	.56
Missing	27 (1.2)	1 (0.1)	NA	NA	5 (0.3)	1 (0.1)	NA	NA
ASA score								
I	271 (12.0)	101 (11.5)	−0.02	<.001	178 (11.5)	101 (11.6)	0.004	.08
II	1327 (58.9)	433 (49.1)	−0.19		856 (55.5)	433 (49.9)	−0.02	
III	635 (28.2)	332 (37.6)	0.2		491 (31.8)	323 (37.3)	0.03	
IV	20 (0.9)	16 (1.8)	0.07		18 (1.2)	10 (1.2)	−0.03	
Missing	27 (1.2)	7 (0.8)	NA		11 (0.7)	7 (0.8)	NA	
Prior surgery or radiation treatment								
Any	429 (19.3)	157 (17.8)	−0.03	.36	285 (18.4)	156 (18.0)	−0.006	.78
Missing	56 (2.5)	6 (0.7)	NA	NA	9 (0.6)	6 (0.7)	NA	NA
Clinical T stage								
CIS/Ta/T1	591 (25.9)	244 (27.4)	0.03	<.001	428 (27.5)	241 (27.6)	0.02	.86
T2	1185 (52.0)	512 (57.6)	0.11		868 (55.9)	501 (57.3)	0.01	
T3	336 (14.7)	86 (9.7)	−0.17		172 (11.1)	85 (9.7)	−0.01	
T4	135 (5.9)	40 (4.5)	−0.07		71 (4.6)	40 (4.6)	0.02	
Unknown^a^	33 (1.4)	7 (0.8)	NA		15 (1.0)	7 (0.8)	NA	
cN+	226 (9.9)	62 (7.0)	−0.12	<.001	123 (7.9)	62 (7.1)	−0.002	.52
NAC								
Any	745 (32.9)	303 (34.2)	0.03	.50	510 (32.9)	297 (34.1)	0.01	.56
Missing	17 (0.7)	3 (0.3)	NA	NA	5 (0.3)	3 (0.3)	NA	NA
Annual hospital volume, procedures/y								
1-10	287 (12.6)	35 (3.9)	−0.45	<.001	65 (4.2)	35 (4.0)	−0.02	.19
11-25	564 (24.7)	144 (16.2)	−0.23		301 (19.4)	144 (16.5)	−0.03	
>25	1429 (62.7)	710 (79.9)	0.43		1188 (76.4)	695 (79.5)	0.02	

Abbreviations: ASA, American Society of Anesthesiologists; BMI, body mass index (calculated as weight in kilograms divided by height in meters squared); CIS, carcinoma in situ; NA, not applicable; NAC, neoadjuvant chemotherapy; ORC, open radical cystectomy; RARC, robot-assisted radical cystectomy; SMD, standardized mean difference.

^a^ Pathologist did not or could not classify a pathological stage.

### Primary Outcomes

**Figure 1.**
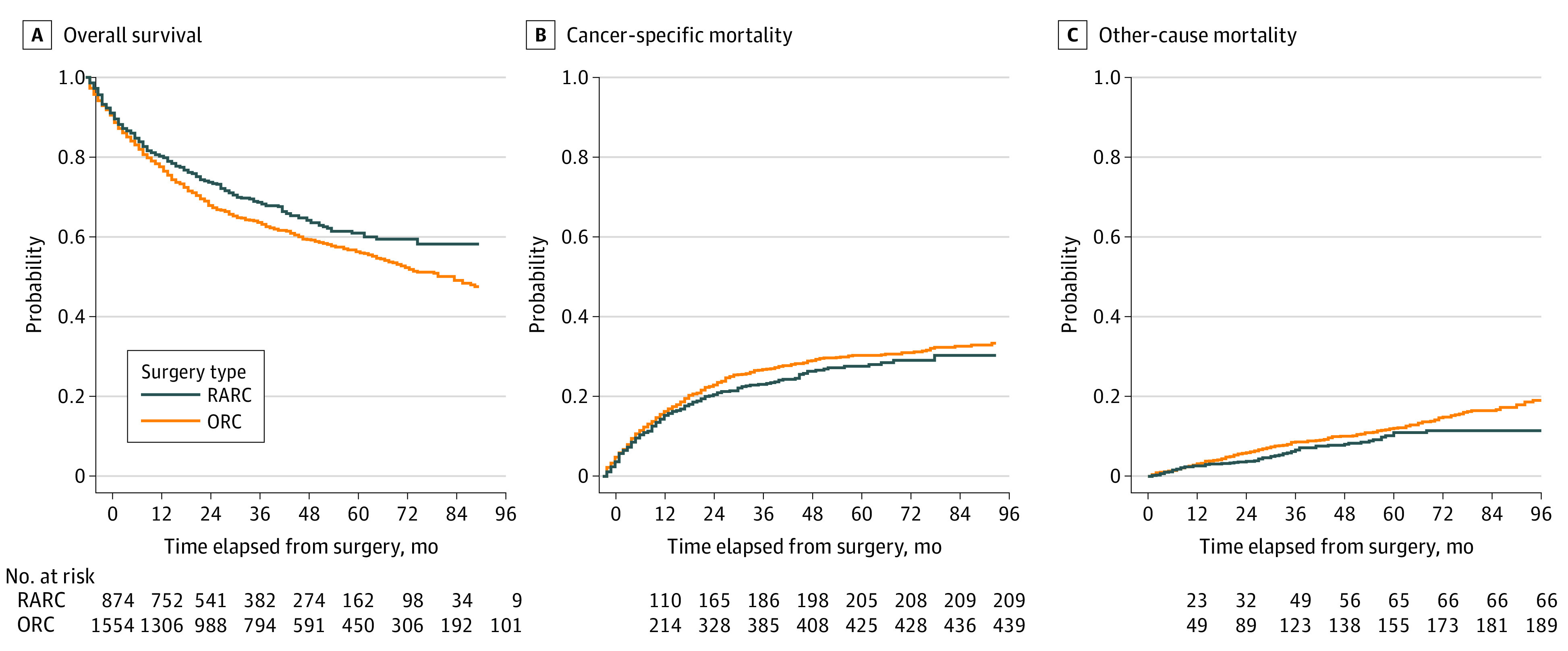
Propensity Score–Matched Survival Analysis ORC indicates open radical cystectomy; RARC, robot-assisted radical cystectomy.

The median (IQR) follow-up time for survivors was 47 (28-71) months (minimum 12 months). Deaths of any cause occurred in 628 patients (40.4%) in the ORC group and 275 patients (31.5%) in the RARC group, and bladder cancer deaths occurred in 439 patients (28.2%) in the ORC group and 209 patients (23.9%) in the RARC group. The estimated cancer-specific mortality rate at 5 years was 30.2% (variance, 1.59) for ORC and 27.6% (variance, 3.12) for RARC, and at 7 years, estimated cancer-specific mortality was 32.3% (variance, 1.91) for ORC and 30.3% (variance, 5.13) for RARC (*P* = .16) (Figure 1). The estimated other-cause mortality rate at 5 years was 12.1% (variance, 0.875) for ORC and 11.0% (variance, 1.99) for RARC, and at 7 years, estimated other-cause mortality was 16.5% (variance, 1.5) for ORC and 11.4% (variance, 2.20) for RARC (*P* = .03) (Figure 1). These differences remained for OS, with 5-year estimates of 57.7% (variance, 2.46) for ORC and 61.4% (variance, 5.11) for RARC, and 7-year estimates of 51.2% (variance, 3.41) for ORC and 58.2% (variance, 7.33) for RARC (*P* = .01) (Figure 1). The 30-day all-cause mortality rate was 1.5% for ORC and 0.9% for RARC (odds ratio [OR], 0.58; 95% CI, 0.15-2.28; *P* = .44), and the 90-day mortality rate was 4.2% for ORC and 2.7% for RARC (OR, 0.70; 95% CI, 0.32-1.54; *P* = .38). In Cox regression analysis, all-cause mortality was superior for RARC (hazard ratio [HR], 0.71; 95% CI, 0.56-0.89; *P* = .004) (eTable 2 in the Supplement). The calculated magnitude of confounding that would be necessary to fully explain the observed risk ratio was *E* = 2.17 (95% CI, not available to 1.50) (eTable 2 in the Supplement). Other parameters independently associated with all-cause mortality were EBL, blood transfusions, higher LN yield, nodal status, and CD grade III or IV complications

To rule out a possible bias of differences in local NAC regimen on survival, a subgroup analysis of patients not receiving systemic therapy before surgery in the matched cohort was performed. All-cause mortality for RARC remained superior compared with ORC (HR, 0.66; 95% CI, 0.49-0.87; *P* = .004).

### Secondary Outcomes

RARC, compared with ORC, was associated with a higher rate of OBS (177 patients [20.3%] vs 116 patients [7.5%]; *P* < .001), lower EBL (median [IQR], 150 [100-300] mL vs 700 [400-1300] mL; *P* < .001), and lower intraoperative transfusion rate (63 patients [7.7%] vs 572 patients [38.7%]; OR, 0.05; 95% CI, 0.03-0.08; *P* < .001) (Table 2). With regard to postoperative outcomes, patients undergoing RARC, compared with those who underwent ORC, had a shorter LOS (median [IQR], 9 [6-13] days vs 13 [10-17] days; *P* < .001) but a higher 90-day rehospitalization rate (294 patients [34.3%] vs 393 patients [26.1%]; OR, 1.28; 95% CI, 1.02-1.60; *P* = .03). LOS was not associated with readmission (OR, 1.00; 95% CI, 0.99-1.01; *P* = .15). The number of removed LNs was significantly higher for RARC than ORC (median [IQR], 20 [15-27] LNs vs 14 [8-24] LNs; *P* < .001).

**Table 2.** Perioperative and Postoperative Characteristics of the PSM Cohort

Characteristic	No. (%)		SMD^a^		*P* value
	ORC (n = 1554)	RARC (n = 874)	Before PSM	After PSM	
**Intraoperative parameters**					
Urethrectomy					
Yes	326 (21.1)	172 (19.8)	NA	NA	.46
Missing	11 (0.7)	5 (0.6)	NA	NA	NA
Deviation type					
Ileum conduit	1386 (89.2)	692 (79.2)	NA	NA	<.001
OBS	116 (7.5)	177 (20.3)	NA	NA	
CCUD	21 (1.4)	1 (0.1)	NA	NA	
Other	31 (2.0)	4 (0.5)	NA	NA	
Missing	0	0	NA	NA	
Estimated blood loss					
Median (IQR), mL	700 (400 to 1300)	150 (100 to 300)	NA	NA	<.001
Missing	19 (1.2)	22 (2.5)	NA	NA	NA
Intraoperative blood transfusion					
Yes	572 (38.7)	63 (7.7)	NA	NA	<.001
Missing	75 (4.8)	59 (6.8)	NA	NA	NA
Perioperative blood transfusion^b^					
Yes	429 (50.8)	143 (20.7)	NA	NA	<.001
Missing	137 (14.0)	12 (1.7)	NA	NA	NA
Operation time					
Median (IQR), min	323 (250 to 407)	320 (260 to 380)	NA	NA	.45
Missing	32 (2.1)	29 (3.3)	NA	NA	NA
**Postoperative parameters**					
Clavien-Dindo classification					
I-II	404 (27.0)	286 (33.3)	NA	NA	.001
III	284 (19.0)	122 (14.2)	NA	NA	
IV	46 (3.1)	19 (2.2)	NA	NA	
V	27 (1.8)	7 (0.8)	NA	NA	
Missing	59 (3.8)	14 (1.6)	NA	NA	
Reoperation					
Yes	182 (12.0)	81 (9.4)	NA	NA	.05
Missing	41 (2.6)	11 (1.3)	NA	NA	NA
Length of stay					
Median (IQR), d	13 (10 to 17)	9 (6 to 13)	NA	NA	<.001
Missing	34 (2.2)	10 (1.1)	NA	NA	NA
Rehospitalization in 90 d					
Yes	393 (26.1)	294 (34.3)	NA	NA	<.001
Missing	48 (3.1)	17 (1.9)	NA	NA	NA
Pathological T-stage					
T0	344 (22.1)	200 (22.9)	0.05	0.001	.99
CIS/Ta/T1	347 (22.3)	198 (22.7)	0.04	0.02	
T2	283 (18.2)	161 (18.4)	0.02	0.02	
T3	418 (26.9)	228 (26.1)	−0.02	−0.01	
T4	151 (9.7)	80 (9.2)	−0.14	−0.02	
Unknown^c^	11 (0.7)	7 (0.8)	NA	NA	
Removed lymph nodes^d^					
Median (IQR), No.	14 (8 to 24)	20 (15 to 27)	NA	NA	<.001
Missing	90 (6.6)	21 (2.6)	NA	NA	NA
N+					
Yes	305 (20.2)	169 (20.4)	NA	NA	.91
Missing	45 (2.9)	46 (5.3)	NA	NA	NA

Abbreviations: CCUD, continent cutaneous urinary diversion; CIS, carcinoma in situ; NA, not applicable; OBS, orthotopic bladder substitution; ORC, open radical cystectomy; PSM, propensity score matching; RARC, robot-assisted radical cystectomy; SMD, standardized mean difference.

^a^ Reported for variables used in propensity score matching.

^b^ Total number of patients receiving transfusions during hospitalization (including intraoperative transfusions). Variable reported since 2014 (after matching, there were 981 patients in the ORC group and 703 in the RARC group).

^c^ Pathologist did not or could not classify a pathological stage.

^d^ In patients undergoing pelvic lymph node dissection.

The overall 90-day complication rate was comparable in ORC and RARC groups (761 patients [50.9%] vs 434 patients [50.5%]) (Table 2). However, RARC was associated with a lower high-grade (CD grade ≥III) complication rate compared with ORC (148 patients [17.2%] vs 357 patients [23.9%]; OR, 0.62; 95% CI, 0.43-0.87; *P* = .009). Infectious complications were more common in the RARC group, while lymphoceles and events concerning the cardiovascular or respiratory system or the abdominal wall or stoma occurred more frequently in the ORC group (Table 3). Differences in categories remained when only high-grade complications were considered, except for infectious complications. The 90-day CD grade V complication rate was 7 patients (0.8%) for the RARC group and 27 patients (1.8%) for the ORC group (OR, 0.73; 95% CI, 0.18-3.00; *P* = .66).

**Table 3.** Subclassifications of Complications

System/category	No. (%)^a^		*P* value
	ORC (n = 1554)	RARC (n = 874)	
**Subclassification of any complication reported**			
Gastrointestinal	192 (12.4)	101 (11.6)	.62
Cardiovascular and respiratory	99 (6.4)	36 (4.1)	.02
Infectious	454 (29.2)	300 (34.4)	.009
Abdominal wall or stoma	187 (12.0)	38 (4.4)	<.001
Genitourinary	115 (7.4)	74 (8.5)	.34
Bleeding	22 (1.4)	16 (1.8)	.50
Lymphocele	65 (4.2)	12 (1.4)	<.001
Other	191 (12.3)	117 (13.4)	.45
**Subclassification of complications in patients with ≥1 high-grade complication (Clavien-Dindo III-V)**			
Gastrointestinal	113 (7.3)	51 (5.8)	.18
Cardiovascular and respiratory	63 (4.1)	14 (1.6)	.001
Infectious	179 (11.6)	87 (10.0)	.22
Abdominal wall or stoma	118 (7.6)	26 (3.0)	<.001
Genitourinary	97 (6.3)	57 (6.5)	.80
Bleeding	18 (1.2)	13 (1.5)	.57
Lymphocele	29 (1.9)	7 (0.8)	.04
Other	72 (4.6)	33 (3.8)	.35

Abbreviations: ORC, open radical cystectomy; RARC, robot-assisted cystectomy.

^a^ Patients can be assigned to multiple categories.

To assess a possible bias of the learning curve on postoperative complications, we compared the rate of CD grade III or greater from 2011 to 2014 vs 2015 to 2018. In this comparison, there was no difference in the ORC group (OR, 0.90; 95% CI, 0.71-1.15; *P* = .36), while for the RARC group, the rate declined significantly (OR, 0.62; 95% CI, 0.43-0.90; *P* = .01).

### Sensitivity Analyses

**Figure 2.**
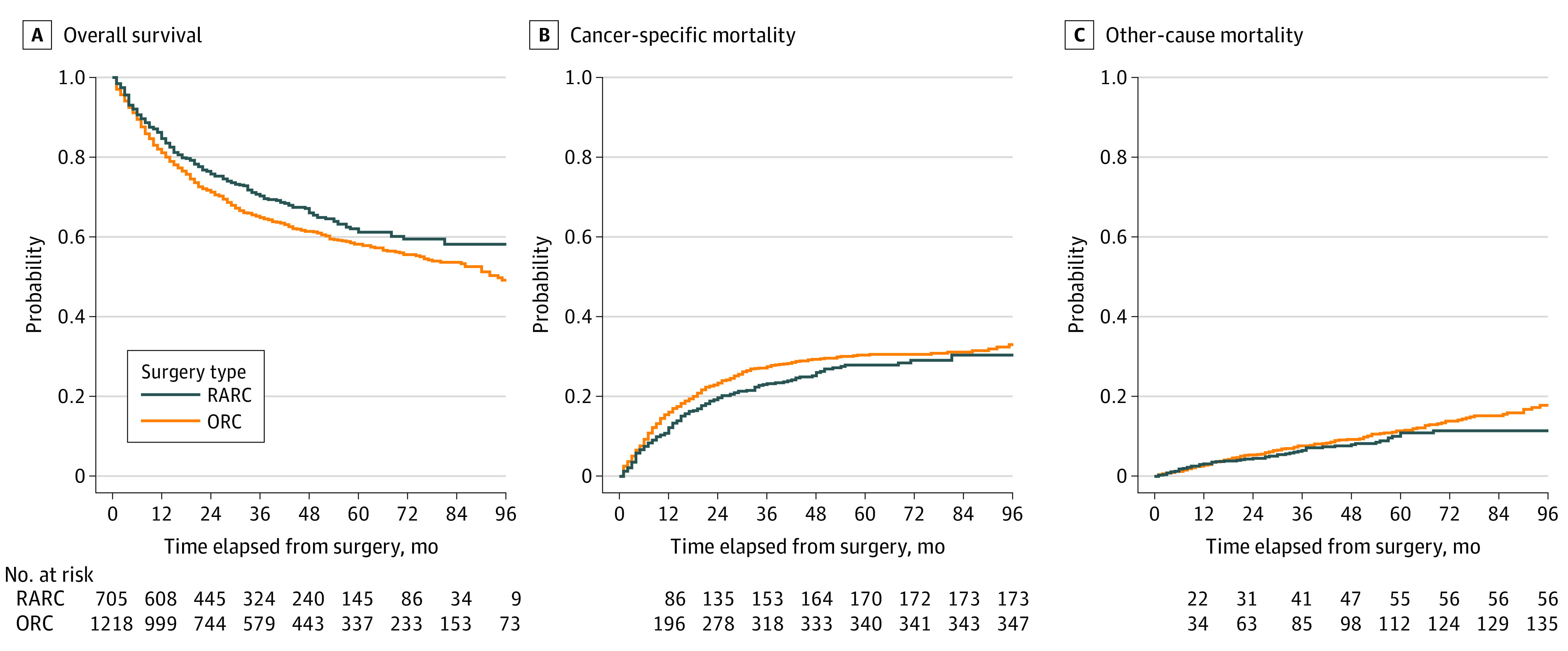
Sensitivity Survival Analysis including High-Volume Hospitals Only Analysis restricted to patients who received care in hospitals with a yearly volume of more than 25 procedures. ORC indicates open radical cystectomy; RARC, robot-assisted radical cystectomy.

Our first sensitivity analysis, including year of surgery as matching variable, showed similar results in regards to the primary and secondary outcomes (eTable 3 and eFigure 3 in the Supplement); therefore, we proceeded with the more parsimonious model that maximized sample size. In the second sensitivity analysis only including high-volume hospitals, the survival and complication advantage of RARC compared with ORC remained, with an estimated 7-year OS of 58.2% (variance, 8.40) in RARC vs 53.7% (variance, 3.97) in ORC (HR, 0.83; 95% CI, 0.71-0.97; *P* = .02) (Figure 2) and a high-grade complications rate (CD grade ≥III) (OR, 0.66; 95% CI, 0.45-0.96; *P* = .03).

## Discussion

In this large cohort study of bladder cancer in Sweden, we found a higher overall mortality after ORC than after RARC. Moreover, the robotic approach had a similar operation time, shorter LOS, and was associated with fewer postoperative high-grade complications. The main strength of this study is the use of a nationwide population-based cohort encompassing nearly all patients with bladder cancer undergoing RC over 8 years, with detailed clinicopathological, perioperative data for long and complete follow-up. We performed a matched analysis to reduce patient selection bias and provide a valid comparison between the 2 techniques. To our knowledge, this is the largest comparative analysis of RARC with ICUD vs ORC controlling for patient, tumor, and hospital characteristics.

Efforts have been made to examine the surgical and oncological safety of RARC compared with the previous standard of care of ORC. Results from 5 prospective randomized clinical trials involving 541 patients have confirmed that RARC and ORC are comparable in terms of complications and oncological outcomes, while RARC reduces the risk of blood transfusions and LOS.^14,15,16,17,18,19,20,21^ However, participants of these trials almost exclusively underwent ECUD.

It has been proposed that ICUD might have potential benefits in terms of decreased fluid loss, further reduced EBL, and quicker return to bowel function.^22,23^ In a 3-way comparison of ORC, RARC with ECUD, and ICUD, the fully intracorporeal approach was associated with reduction of EBL, LOS, ileus rate, and major complication rates but longer operation time compared with the other 2 techniques.^24^ However, studies investigating the potential benefits of ICUD are limited to single-institution series with limited sample sizes.^25^ Our findings suggest superior perioperative outcomes within the RARC cohort; we found that RARC with ICUD was associated with an 80% lower EBL and blood transfusion rate. While the median EBL of 700 mL for ORC in this study was in line with other reported rates for ORC (median [IQR], 800 [600-1150] mL), the median EBL of 150 mL for RARC with ICUD was significantly lower than reported volumes for RARC with ECUD (median [IQR], 350 [288-453] mL).^25^ This difference further translated into a significantly lower intraoperative transfusion rate of only 8% vs 39% for ORC.

While the overall complication rate was comparable between groups, we observed a significantly lower high-grade complication rate in the RARC cohort. In line with prior reports^17,19,20^ the overall infectious complication rate was significantly higher for RARC compared with ORC. However, this difference of 5.2% diminished in the subanalysis of patients with high-grade complications only. At the same time, complications concerning the cardiovascular or respiratory system, the abdominal wall or stoma, and lymphoceles remained more frequent in the ORC group, suggesting that these kind of incidents were more likely to be CD graded as III or greater than infectious complications. While the 23.9% rate of CD grade III or greater in the ORC group was comparable with that of ORC cohorts in other comparative studies (20%-22%) the rate for RARC with ICUD was 17.2%, lower than reported rates for RARC with ECUD (22%-35%).^15,17,20^ However, owing to the technically more challenging nature of ICUD, a higher complication rate at the beginning of the learning curve, as observed in this study, has to be taken into consideration.^26^

While LOS was shorter in the RARC group compared with the ORC group, the readmission rate was significantly higher. We investigated if an earlier postoperative discharge, attributed to an assumed broader implementation of enhanced recovery after surgery protocols at RARC centers, may have contributed to increased readmissions. However, this was not the case (OR, 1.00; 95% CI, 0.99-1.01; *P* = .15). Although the reason for rehospitalization was not available, we hypothesize that the higher rate of infectious complications in the RARC cohort, which in many cases make intravenous antibiotics necessary based on local guidelines, may have attributed to this finding.

A meticulous PLND is an integral part of RC, although the extent of the dissection is controversial.^27^ Prior studies have reported equal^15^ or higher^28^ LN yields for RARC, contradicting concerns regarding the quality of PLND for this technique. In this study, the median LN yield was significantly higher for RARC compared with ORC. Notably, the higher LN yield did not prolong the operation time. Moreover, we observed a comparable operation time in the RARC group, in spite of a higher rate of OBS. Although the SNRUBC database did not contain information on conversion, these findings add more evidence that after moving forward on the learning curve, RARC with high quality PLND and ICUD can be performed in a favorable time frame. The higher rate of OBS in the RARC group remained after adjusting for known factors associated with the choice of urinary diversion, such as age, tumor stage, and prior surgery and radiation therapy. It has been shown that OBS are constructed more frequently at academic centers and even at high-volume centers, this rate varies widely among institutions suggesting a substantial influence of the surgeon or local guidelines.^29^ Indeed, the largest national hospital predominantly contributing to the RARC cohort was an academic center and had also the highest rate for OBS.

Initially, the question had been raised of whether RARC would be associated with worse survival outcomes, potentially owing to lower LN yields or alteration of recurrence patterns due to tumor seeding linked to the pneumoperitoneum or insufflation.^30^ Meanwhile, randomized clinical trials have demonstrated the noninferiority of RARC with ECUD in terms of progression-free, cancer-specific, and overall survival.^21^ However, long-term oncological follow-up data for RARC with ICUD alone in a representative cohort or in comparison with ORC have not been reported, to our knowledge.^31^ We observed a significantly higher OS rate for patients undergoing RARC compared with ORC, but there was no difference in cancer-specific mortality. The OS benefit was 2% in the first year and increased to around 7% after 7 years. The improved OS rate for RARC remained after adjusting for the year of surgery and excluding low-volume hospitals. We assume that a combination of multiple factors of the minimal invasive approach may have contributed to the OS benefit detected in this study. Besides the observed lower CD V complication rate for RARC, EBL, blood transfusions, higher LN yield, and CD grade III or IV complications were associated with all-cause mortality in our Cox analysis; RARC with ICUD had a beneficial association with all of them (eTable 2 in the Supplement).

A central concern with use of observational data is bias by unmeasured confounding. To address this issue, we calculated *E*-values for the mortality estimates. Using the *E*-values, we found that an unmeasured confounder needed to be associated with all-cause mortality and surgical approach by a risk ratio of 2.17 to explain away the observed risk ratio of 0.71 in favor of RARC. While we cannot rule out the presence of such an unmeasured confounder, any uncontrolled confounder sufficient to cancel our results would also have to be independent of the multiple covariates already adjusted for in the analysis, which is unlikely to be the case.

### Limitations

This study has several limitations. As in all register-based studies, misclassification and underreporting are potential concerns. PS methods adjust for known confounders but do not adjust for unobserved differences between the groups with potential associations with survival outcome, which is an inherent limitation of a retrospective analysis. Notably, specific data for comorbidities were not available. Nevertheless, age and ASA score were well balanced between the groups after PS matching, with initially more RARC patients in ASA categories III and IV. The variability among the institutions in terms of surgical techniques, perioperative protocols (eg, enhanced recovery after surgery), and pathology reporting represents another limitation. Information on whether cystectomies were performed for curative, salvage, or palliative reasons was not captured in the SNRUBC database. Similarly, data on surgeon experience, conversion rate, histological subtypes, surgical margin status, and detailed information about the type or frequency of NAC are lacking. To overcome some of these limitations, a prospective trial with estimated completion by the end of 2021 is in progress: iROC, a multicenter randomized clinical trial comparing RARC with ICUD vs ORC.^32^ Although, more follow-up time will be needed to assess survival, iROC will provide level 1 evidence for perioperative outcome and complications.

## Conclusions

In this cohort study, we observed a lower high-grade complication rate and a significant OS benefit for RARC compared with ORC. The minimally invasive method was associated with lower EBL, lower intraoperative transfusion rate, higher LN yield, and shorter LOS compared with the open technique. However, the 90-day readmission rate was higher. In spite of the technically complex nature of the fully intracorporeal approach predominantly performed in Sweden, our findings support the feasibility of a nationwide implementation with beneficial outcomes for patients with bladder cancer.

## Notes

Supplement. **eFigure 1.** Survival Analysis Before Propensity Score Matching **eTable 1.** Univariate Cox Regression Model Before Propensity Score Matching **eFigure 2.** Propensity Score Distributions Before and After Propensity Score Matching **eTable 2.** Univariable Cox Regression Analysis and E-Values for Mortality Analysis After Propensity Score Matching **eTable 3.** Sensitivity Analysis for Year of Surgery, Baseline Characteristic, Outcomes, Univariable Cox Regression Analysis for All-Cause Mortality **eFigure 3.** Sensitivity Survival Analysis for Year of Surgery
