# Supplementary material for: Association of Open vs Robot-Assisted Radical Cystectomy With Mortality and Perioperative Outcomes Among Patients With Bladder Cancer in Sweden
Source: JAMA Netw Open. 2022 Apr 28;5(4):e228959. doi: 10.1001/jamanetworkopen.2022.8959 (PMC9051984; doi:10.1001/jamanetworkopen.2022.8959)
Supplement: Supplement. — eFigure 1. Survival Analysis Before Propensity Score Matching eTable 1. Univariate Cox Regression Model Before Propensity Score Matching eFigure 2. Propensity Score Distributions Before and After Propensity Score Matching eTable 2. Univariable Cox Regression Analysis and E-Values for Mortality Analysis After Propensity Score Matching eTable 3. Sensitivity Analysis for Year of Surgery, Baseline Characteristic, Outcomes, Univariable Cox Regression Analysis for All-Cause Mortality eFigure 3. Sensitivity Survival Analysis for Year of Surgery [file jamanetwopen-e228959-s001.pdf]

Supplemental Online Content

Mortezavi A, Crippa A, Kotopouli MI, Akre O, Wiklund P, Hosseini A. Association of open vs robot-assisted radical cystectomy with mortality and perioperative outcomes among patients with bladder cancer in Sweden. *JAMA Netw Open*. 2022;5(4):e228959. doi:10.1001/jamanetworkopen.2022.8959

eFigure 1. Survival Analysis Before Propensity Score Matching
eTable 1. Univariate Cox Regression Model Before Propensity Score Matching
eFigure 2. Propensity Score Distributions Before and After Propensity Score Matching
eTable 2. Univariable Cox Regression Analysis and E-Values for Mortality Analysis After Propensity Score Matching
eTable 3. Sensitivity Analysis for Year of Surgery, Baseline Characteristic, Outcomes, Univariable Cox Regression Analysis for All-Cause Mortality
eFigure 3. Sensitivity Survival Analysis for Year of Surgery

This supplemental material has been provided by the authors to give readers additional information about their work.

© 2022 Mortezavi A et al. *JAMA Network Open.*

eFigure 1. Survival Analysis Before Propensity Score Matching

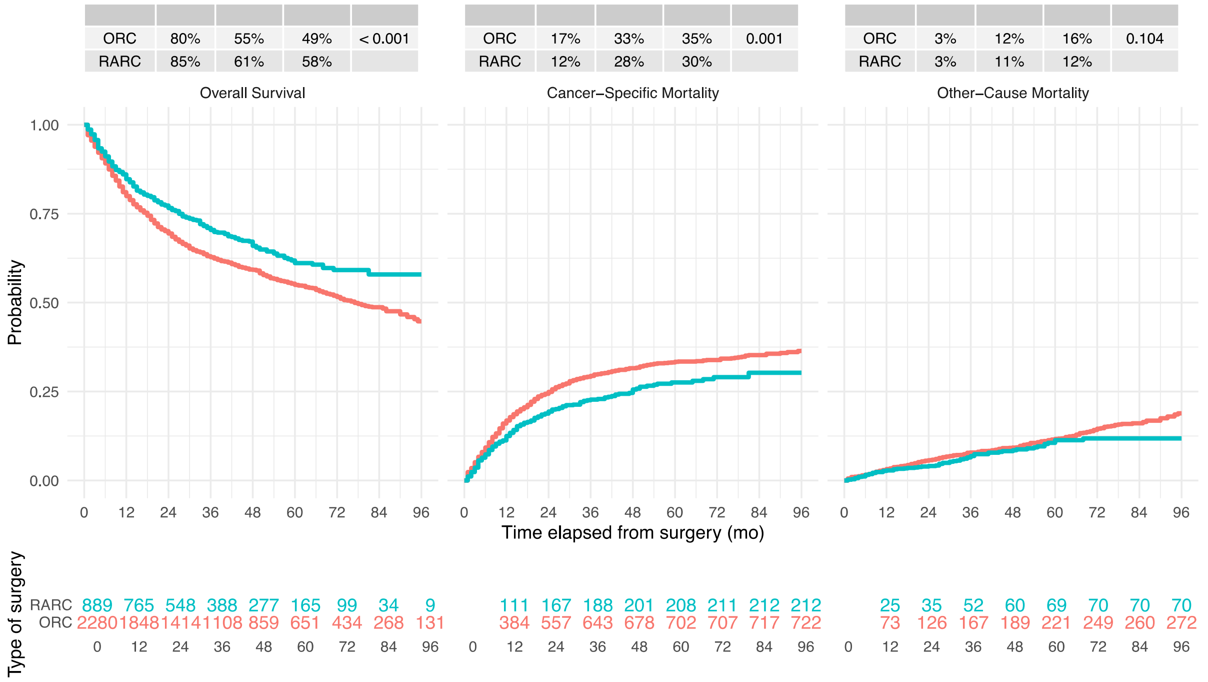

Cumulative incidence functions illustrating cancer-specific (CSM), other-cause mortality (OCM), and overall survival (OS, 1 – all-cause mortality) for patients undergoing open radical cystectomy (ORC) and robot-assisted radical cystectomy (RARC). Tables include the number of events for CSM and OCM, numbers at risk for OS, and estimates for 1, 5 and 7 years. P-value using the Gray's and log-rank tests.

© 2022 Mortezavi A et al. *JAMA Network Open.*

eTable 1. Univariate Cox Regression Model Before Propensity Score Matching

	**Characteristics**	**All-cause mortality**		
		**HR**	**95% CI**	**p***
Female gender	female vs. male	1.05	0.93-1.19	0.43
Age	years, continious (interval 5)	1.17	1.13-1.21	**<0.001**
BMI	kg/m², continious (interval 5)	0.84	0.79-0.90	**<0.001**
ASA score	III/IV vs. I/II	1.62	1.44-1.81	**<0.001**
Prior surgery / RTx	yes vs. no	1.12	0.98-1.28	0.12
Clinical T stage	cT2 vs. Ta/T1/Tis	1.77	1.52-2.05	**<0.001**
	cT3 vs. Ta/T1/Tis	2.41	2.00-2.90	**<0.001**
	cT4 vs. Ta/T1/Tis	3.15	2.50-3.97	**<0.001**
Clinical N stage	cN+ vs. cN-	1.76	1.49-2.08	**<0.001**
NAC	yes vs. no	0.77	0.68-0.87	**<0.001**
Annual hospital volume	11-25 vs. 1-10	0.82	0.68-0.99	**0.04**
	>25 vs. 1-10	0.79	0.67-0.93	**0.006**
Pathological stage	pT2 vs. pT0/pTa/T1/Tis	1.99	1.66-2.38	**<0.001**
	pT3 vs. pT0/pTa/T1/Tis	4.13	3.56-4.79	**<0.001**
	pT4 vs. pT0/pTa/T1/Tis	7.6	6.43-8.98	**<0.001**

BMI: body mass index, ASA: American Society of Anesthesiologists, RTx: radio therapy, NAC: neoadjuvant chemotherapy, HR: hazard ratio, CI: confidence interval. * Bold face reprensenting P < 0.05.

© 2022 Mortezavi A et al. *JAMA Network Open.*

eFigure 2. Propensity Score Distributions Before and After Propensity Score Matching

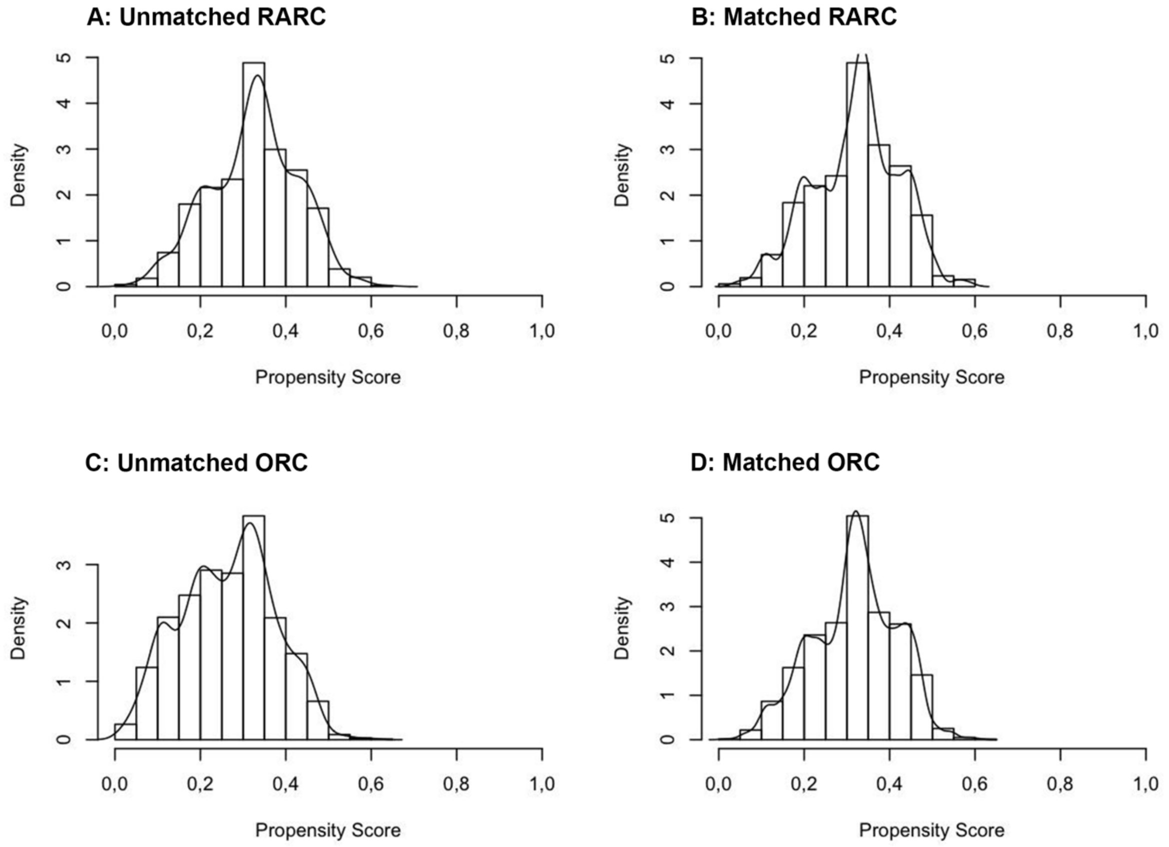

The propensity score distributions for the groups both before and after matching; Figure 1A and C demonstrate an initial dissimilarity on the basis of propensity score distributions. After matching, the distributions of the propensity scores (Figure 1 B and D) were homogeneous and confirmed the adequacy of the propensity score model to achieve balance. RARC: robot-assisted radical cystectomy, ORC: open radical cystectomy

eTable 2. Univariable Cox Regression Analysis and E-Values for Mortality Analysis After Propensity Score Matching

eTable 2 (A). Univariable Cox regression analysis for all-cause, cancer-specific, and other-cause mortality (after matching)

		Before PSM			After PSM								
**Variable**	**Characteristics**	**All-cause mortality**			**All-cause mortality**			**Cancer-specific mortality**			**Other-cause mortality**		
		**HR**	**95% CI**	**p***	**HR**	**95% CI**	**p***	**HR**	**95% CI**	**p***	**HR**	**95% CI**	**p***
Estimated blood loss	continuous, ml (per 250)	1.04	1.02-1.05	**<0.001**	1.03	1.01-1.04	**0.001**	1.02	1.00-1.04	**0.015**	1.03	1.01-1.06	**0.012**
Blood transfusion	yes vs. no	1.63	1.44-1.85	**<0.001**	1.69	1.45-1.97	**<0.001**	1.71	1.42-2.05	**<0.001**	1.64	1.25-2.16	**<0.001**
No. of removed LN*	continious (interval 5)	0.92	0.90-0.95	**<0.001**	0.90	0.86-0.94	**<0.001**	0.91	0.86-0.95	**<0.001**	0.88	0.82-0.95	**<0.001**
Nodal status	N+ vs. N-	3.11	2.77-3.50	**<0.001**	3.28	2.85-3.78	**<0.001**	3.97	3.39-4.66	**<0.001**	1.79	1.33-2.42	**<0.001**
Complications	CD I-II vs. none	1.00	0.87-1.14	0.97	1.03	0.88-1.22	0.69	1.09	0.90-1.31	0.383	0.89	0.65-1.22	0.460
	CD III vs. none	1.21	1.04-1.41	**0.013**	1.26	1.04-1.51	**0.016**	1.20	0.96-1.50	0.105	1.39	1.01-1.91	**0.041**
	CD IV vs. none	1.88	1.43-2.47	**<0.001**	1.71	1.18-2.50	**0.005**	1.28	0.78-2.12	0.329	2.76	1.54-4.95	**0.001**
Type of surgery	RARC vs. ORC	0.81	0.70-0.93	**0.003**	0.71	0.56-0.89	**0.004**	0.77	0.58-1.01	0.057	0.57	0.37-0.89	**0.013**

*PSM: propensity score matching, LN: lymph nodes, CD: Clavien-Dindo, RARC: robot-assisted radical cystectomy, ORC: open radical cystectomy, HR: hazard ratio, CI: confidence interval. Bold face reprensenting P < 0.05. * in patients undergoing pelvic lymph node dissection*

© 2022 Mortezavi A et al. *JAMA Network Open.*

eTable 2 (B). E-Values for all-cause, cancer-specific, and other-cause mortality (in the matched cohort)

Variable	Characteristics	All-cause mortality		Cancer-specific mortality		Other-cause mortality	
		E-value	95% CI	E-value	95% CI	E-value	95% CI
Estimated blood loss	continuous, ml (per 250)	1.21	1.11-NA	1.16	1.00-NA	1.21	1.11-NA
Blood transfusion	yes vs. no	2.77	2.26-NA	2.81	2.19-NA	2.66	1.81-NA
No. of removed LN*	continious (interval 5)	1.46	NA-1.32	1.43	NA-1.29	1.53	NA-1.29
Nodal status	N+ vs. N-	6.01	5.15-NA	7.40	6.24-NA	2.98	1.99-NA
Complications	CD I-II vs. none	1.21	1.00-NA	1.40	1.00-NA	1.50	NA-1.00
	CD III vs. none	1.83	1.24-NA	1.69	1.00-NA	2.13	1.11-NA
	CD IV vs. none	2.81	1.64-NA	1.88	1.00-NA	4.96	2.45-NA
Type of surgery	RARC vs. ORC	2.17	NA-1.50	1.92	NA-1.00	2.90	NA-1.50

*LN: lymph nodes, CD: Clavien-Dindo, RARC: robot-assisted radical cystectomy, ORC: open radical cystectomy, CI: confidence interval. * in patients undergoing pelvic lymph node dissection. NA: not applicable*

© 2022 Mortezavi A et al. *JAMA Network Open.*

eTable 3. Sensitivity Analysis for Year of Surgery, Baseline Characteristic, Outcomes, Univariable Cox Regression Analysis for All-Cause Mortality

eTable 3 (A): Sensitivity analysis for year of surgery, baseline characteristics							
		**Before propensity matching**			**After propensity matching**		
		**ORC (n=2280)**	**RARC (n=889)**	**SMD***	**ORC (n=1457)**	**RARC (n=879)**	**SMD***
Female gender, n (%)		567 (24.9)	216 (24.3)	-0.01	339 (23.3)	213 (24.2)	-0.02
Age (y)		71 (66, 76)	71 (65, 76)	-0.07	71 (65, 76)	71 (65, 76)	-0.01
BMI (kg/m2)		26 (23, 28)	26 (23, 28)	0.21	26 (23, 28)	26 (23, 28)	-0.01
	Missing	27 (1.2)	1 (0.1)		4 (0.3)	1 (0.1)	
ASA score, n (%)							
	I	271 (12.0)	101 (11.5)	-0.02	170 (11.8)	100 (11.5)	-0.02
	II	1327 (58.9)	433 (49.1)	-0.19	778 (53.8)	433 (49.7)	-0.01
	III	635 (28.2)	332 (37.6)	0.2	479 (33.1)	326 (37.4)	0.02
	IV	20 (0.9)	16 (1.8)	0.07	18 (1.2)	13 (1.5)	0.001
	Missing	27 (1.2)	7 (0.8)		12 (0.8)	7 (0.8)	
Prior surgery / RTx, n (%)		429 (19.3)	157 (17.8)	-0.03	278 (19.3)	154 (17.6)	-0.01
	Missing	56 (2.5)	6 (0.7)		13 (0.9)	6 (0.7)	
Clinical T stage, n (%)							
	Tis/Ta/T1	591 (25.9)	244 (27.4)	0.03	413 (28.3)	241 (27.4)	-0.02
	T2	1185 (52.0)	512 (57.6)	0.11	788 (54.1)	505 (57.5)	0.03
	T3	336 (14.7)	86 (9.7)	-0.17	166 (11.4)	86 (9.8)	-0.01
	T4	135 (5.9)	40 (4.5)	-0.07	76 (5.2)	40 (4.6)	-0.01
	cTx	33 (1.4)	7 (0.8)		14 (1.0)	7 (0.8)	
Clinical N stage, n (%)							
	cN+	226 (9.9)	62 (7.0)	-0.12	121 (8.3)	62 (7.1)	-0.02
NAC, n (%)		745 (32.9)	303 (34.2)	0.03	493 (33.9)	300 (34.2)	-0.02
	Missing	17 (0.7)	3 (0.3)		4 (0.3)	3 (0.3)	
Annual hospital vol., n (%)							
	1 - 10	287 (12.6)	35 (3.9)	-0.45	58 (4.0)	35 (4.0)	-0.01
	11 - 25	564 (24.7)	144 (16.2)	-0.23	286 (19.6)	144 (16.4)	-0.01
	>25	1429 (62.7)	710 (79.9)	0.43	1113 (76.4)	700 (79.6)	-0.007
Year of surgery							
	2011-2013	865 (37.9)	173 (19.5)	-0.47	360 (24.7)	173 (19.7)	-0.01
	2014-2015	597 (26.2)	255 (28.7)	0.06	422 (29.0)	251 (28.6)	0.02
	2016-2018	818 (35.9)	461 (51.9)	0.32	675 (46.3)	455 (51.8)	-0.003

Data presented as median (quartile 1, quartile 3) or frequency (percentage). ORC: open radical cystectomy, RARC: robot-assisted radical cystectomy, SMD: standartized mean difference, y: year, BMI: body mass index, ASA: American Society of Anesthesiologists, RTx: radio therapy, NAC: neoadjuvant chemotherapy, vol.: volume.

© 2022 Mortezavi A et al. *JAMA Network Open.*

eTable 3 (B): Sensitivity analysis for year of surgery, outcome parameters					SMD	
		ORC (n=1457)	RARC (n=879)	p	before PSM*	after PSM*
**INTRAOPERATIVE PARAMETERS**						
Urethrectomy , n (%)		290 (20.0)	170 (19.5)	0.8		
	Missing	7 (0.5)	5 (0.6)			
Deviation type, n (%)						
	Ileum conduit	1299 (89.2)	694 (79.0)	<0.001		
	OBS	103 (7.1)	180 (20.5)			
	CCUD	20 (1.4)	1 (0.1)			
	Other	34 (2.3)	4 (0.5)			
	Missing	1 (0.1)	0 (0.0)			
Estimated blood loss (ml)		700 (400, 1200)	150 (100, 300)	<0.001		
	Missing	21 (1.4)	22 (2.5)			
Intra-OP blood transfusion, n (%)		512 (37.2)	63 (7.7)	<0.001		
	Missing	82 (5.6)	59 (6.7)			
Peri-OP blood transfusion**, n (%)		478 (50.7)	144 (20.7)	<0.001		
	Missing	155 (14.1)	12 (1.7)			
Operation time (min)		320 (244, 420)	320 (260, 380)	0.5		
	Missing	24 (1.6)	29 (3.3)			
**POSTOPERATIVE PARAMETERS**						
Clavien-Dindo classification, n (%)						
	I-II	374 (26.4)	283 (32.7)	0.001		
	III	264 (18.6)	123 (14.2)			
	IV	50 (3.5)	19 (2.2)			
	V	21 (1.5)	7 (0.8)			
	Missing	41 (2.8)	14 (1.6)			
Re-operation, n (%)		177 (12.4)	82 (9.4)	0.03		
	Missing	33 (2.3)	11 (1.3)			
Lenghts of stay (days)		13 (10, 17)	9 (6, 13)	<0.001		
	Missing	37 (2.5)	10 (1.1)			
Re-hospitalization in 90d, n (%)		377 (26.8)	291 (33.8)	<0.001		
	Missing	50 (3.4)	17 (1.9)			
Pathological T-stage, n (%)						
	T0	335 (23.0)	200 (22.8)	0.9	0.05	-0.01
	Tis/Ta/T1	321 (22.0)	201 (22.9)		0.04	0.01
	T2	260 (17.8)	161 (18.3)		0.02	0.003
	T3	377 (25.9)	230 (26.2)		-0.02	0.01
	T4	153 (10.5)	80 (9.1)		-0.14	-0.03
	pTx	11 (0.8)	7 (0.8)			
Removed lymph nodes, n (%)***		14 (8, 25)	20 (15, 27)	<0.001		
	Missing	85 (6.6)	23 (2.8)			
N+, n (%)		279 (19.7)	171 (20.6)	0.6		
	Missing	42 (2.9)	48 (5.5)			

Data presented as median (quartile 1, quartile 3) or frequency (percentage). ORC: open radical cystectomy, RARC: robot-assisted radical cystectomy, SMD: standartized mean difference, PLND: pelvic lymh node diessection, OBS: orthotopic bladder substitution, CCUD:

© 2022 Mortezavi A et al. *JAMA Network Open.*

continent cutaneous urinary diversion, LN: lymph node. *Reported for variables used in propensity score matching, ** Total no of patients receiving transfusions during hospitalisation (including intra-OP transfusions). Variable reported since 2014 (denominator post matching ORC n=1097 and RARC n=706), ***in patients undergoing PLND.

RARC was associated with a lower high-grade complication (CD ≥III) rate compared with ORC (17.2% vs. 23.6%, OR=0.67, 95% CI: 0.54-0.83; p<0.001).

eTable 3 (C): Sensitivity analysis for year of surgery, univariable Cox regression analysis for all-cause mortality

**Variable**	**Characteristics**	**All-cause mortality**		
		**HR**	**95% CI**	**p***
Estimated blood loss	continuous, ml (per 250)	1.03	1.02-1.05	<0.001
Blood transfusion	yes vs. no	1.76	1.53-2.03	<0.001
No. of removed LN*	continious (interval 5)	0.90	0.88-0.93	<0.001
Nodal status	N+ vs. N-	3.34	2.90-3.85	<0.001
Complications	CD I-II vs. none	1.00	0.84-1.18	0.983
	CD III vs. none	1.22	1.00-1.47	0.044
	CD IV vs. none	2.24	1.62-3.11	<0.001
Type of surgery	RARC vs. ORC	0.83	0.72-0.96	0.011

LN: lymph nodes, CD: Clavien-Dindo, RARC: robot-assisted radical cystectomy, ORC: open radical cystectomy, HR: hazard ratio, CI: confidence interval. Bold face reprensenting P < 0.05.
** in patients undergoing pelvic lymph node dissection*

© 2022 Mortezavi A et al. *JAMA Network Open.*

eFigure 3. Sensitivity Survival Analysis for Year of Surgery

ORC	82%	58%	53%	0.011
RARC	85%	61%	58%	

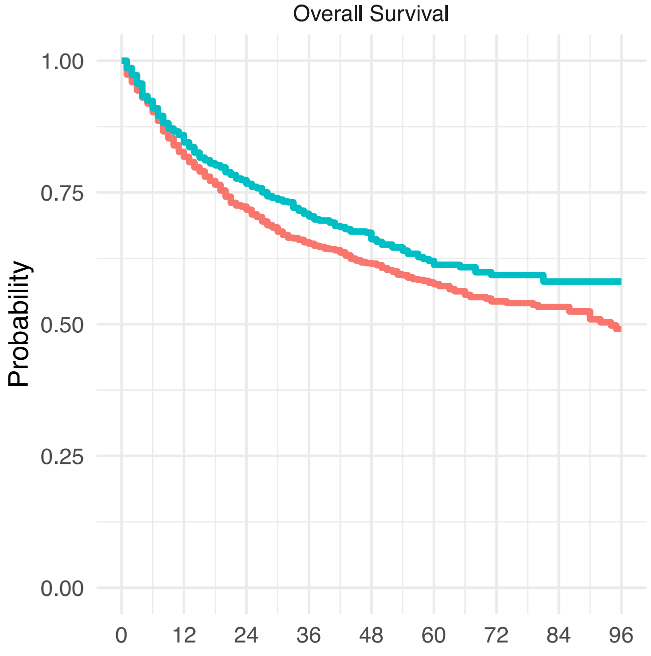

Type of surgery	0	12	24	36	48	60	72	84	96
RARC	879	755	543	384	276	165	99	34	9
ORC	1457	1205	887	668	484	342	184	126	69

Cumulative incidence functions illustrating overall survival (OS) for patients undergoing open radical cystectomy (ORC) and robot-assisted radical cystectomy (RARC). Tables include the numbers at risk for OS, and estimates for 1, 5 and 7 years. P-value using log-rank tests.

Due to a slightly higher rate of pT4 tumors in the ORC group (10.5% vs. 9.1%) after propensity score matching including the year of surgery, we performed a subgroup analysis and excluded all patients with pT4 tumor. The difference in OS remained statistically significant with a benefit for RARC (p=0.015)

© 2022 Mortezavi A et al. *JAMA Network Open.*
